# Determinants of malignant pleural mesothelioma survival and burden of disease in France: a national cohort analysis

**DOI:** 10.1002/cam4.1378

**Published:** 2018-02-26

**Authors:** Christos Chouaid, Jean Baptiste Assié, Pascal Andujar, Cecile Blein, Charlène Tournier, Alexandre Vainchtock, Arnaud Scherpereel, Isabelle Monnet, Jean Claude Pairon

**Affiliations:** ^1^ GRC OncoThoParisEst Service de Pneumologie, CHI Créteil UPEC Créteil France; ^2^ Inserm U955 Institut Santé Travail Paris Est Service de Pneumologie et de Pathologie Professionnelle CHI Créteil Créteil France; ^3^ HEVA Lyon France; ^4^ Thoracic Oncology Department University of Lille CHU Lille, CIIL, Inserm U1019 Créteil France

**Keywords:** Costs, management, mesothelioma, outcomes, social deprivation

## Abstract

This study was undertaken to determine the healthcare burden of malignant pleural mesothelioma (MPM) in France and to analyze its associations with socioeconomic deprivation, population density, and management outcomes. A national hospital database was used to extract incident MPM patients in years 2011 and 2012. Cox models were used to analyze 1‐ and 2‐year survival according to sex, age, co‐morbidities, management, population‐density index, and social deprivation index. The analysis included 1,890 patients (76% men; age: 73.6 ± 10.0 years; 84% with significant co‐morbidities; 57% living in urban zones; 53% in highly underprivileged areas). Only 1% underwent curative surgical procedure; 65% received at least one chemotherapy cycle, 72% of them with at least one pemetrexed and/or bevacizumab administration. One‐ and 2‐year survival rates were 64% and 48%, respectively. Median survival was 14.9 (95% CI: 13.7–15.7) months. The mean cost per patient was 27,624 ± 17,263 euros (31% representing pemetrexed and bevacizumab costs). Multivariate analyses retained men, age >70 years, chronic renal failure, chronic respiratory failure, and never receiving pemetrexed as factors of poor prognosis. After adjusting the analysis to age, sex, and co‐morbidities, living in rural/semi‐rural area was associated with better 2‐year survival (HR: 0.83 [95% CI: 0.73–0.94]; *P* < 0.01); social deprivation index was not significantly associated with survival. With approximately 1,000 new cases per year in France, MPMs represents a significant national health care burden. Co‐morbidities, sex, age, and living place appear to be significant factors of prognosis.

## Introduction

Malignant pleural mesothelioma (MPM) is a rare and aggressive tumor. Because it is mainly associated with asbestos exposure, its incidence varies among countries and population subgroups, depending on the degree of exposure 1, 2. Approximately 2500 new cases are diagnosed annually in the United States and approximately 5000 in Western Europe. MPM incidence is continuously increasing in some countries, like Australia and the United Kingdom 2, 3, 4, but has remained very stable for over 5 years in the United States and Japan. In France, the epidemiological pattern is different because asbestos use started being strictly controlled as early as 1978 3, 4. New cases are still diagnosed due to the long latency of disease onset but, apparently, the incidence in men peaked in 2000–2005. However, since then, the incidence continues to rise in women, for whom professional exposure is often unknown. The number of MPM‐related deaths is approximately 1100/year for men and around 300/year for women in France, while the incidence ranges from less than 1/million of the general population to 50–100/million for at‐risk subgroups 4.

Extra‐pleural pneumonectomy preceded by neoadjuvant chemotherapy and followed by hemithorax irradiation has almost been abandoned in routine practice in France 5, 6, 7. Treatment of advanced MPMs relies primarily on chemotherapy with combination platinum and pemetrexed 8, 9, 10 and, more recently, bevacizumab 11. No oncogenic driver has been identified and molecular pathways leading to MPM have also not yet been clearly determined. To date, no evidence supports using specific targeted therapies to treat MPMs, and most clinical trials yielded negative outcomes.

France has a universal healthcare system largely financed by public health insurance funds. In theory, there are no financial barriers to access to health services, and sociological and geographic parameters should play only a marginal role in MPM outcomes (i.e., survival). Nonetheless, data concerning such influences on MPM management are very limited. Also, few published data are available on the medical costs of MPM management 12, 13, 14. The objectives of this study were to assess the healthcare burden of MPM and investigate potential associations between socioeconomic deprivation and urbanization with MPM incidence and survival in France.

## Methods

### Study design

This retrospective, longitudinal analysis used data extracted from the French National Hospital database (Diagnosis‐Related Groups, DRGs) for all hospitalizations, crossed with geographically aggregated socioeconomic variables at the smallest, most local administrative locality in France(*commune*), documented in the national census databases of the French National Statistics Office (INSEE) through individual patient postal codes.

### DRG database

The DRG covers all public‐ and private‐sector hospitalizations involving short‐term stays in medical, surgical, or obstetric facilities, representing >95% of all hospitalizations in France. The reasons for hospitalization are coded by ICD‐10 diagnosis 15, either as principal diagnoses (PD), related diagnoses (RD: any underlying condition which may have been related to the PD) or as significantly‐associated diagnoses (SAD; co‐morbidities which may affect the course or cost of hospitalization). Demographic data is limited to age, sex, and home‐address postal code. Patients can be tracked across multiple hospitalizations through a unique anonymous patient identifier, which is conserved until the patient dies.

### Study population

The analysis included all patients with a documented ICD‐10 code for MPM (C450, C459) as PD, RD, or SAD for any hospital stay in 2011 and 2012 and, to restrict the sample to incident cases, without an ICD‐10 code for MPM since 2006 (i.e.,: no hospitalization for MPM before 2011). This inclusion period was chosen to allow follow‐up of all patients for at least 2 years. For each patient, information was documented on sex, age at diagnosis, type of hospital where the patient was first admitted for MPM management, concomitant chronic co‐morbidities (hypertension, diabetes mellitus, renal insufficiency, chronic obstructive pulmonary disease (COPD), pulmonary insufficiency and/or other chronic lung disease(s)), and survival status at 1 and 2 years.

Each patient's administrative residence locality was determined from his/her postal code. A commune generally consists of a single locality and any surrounding hamlets or countryside, with a typical area of 10–50 km^2^. Data on each locality's socio‐demographic composition were retrieved from the French National Statistics Office and used to classify it in terms of population density and social deprivation. Based on national census data**,** population density was divided into four classes based on its number of inhabitants: rural (<2000), semi‐rural (2000–9999), semi‐urban (10,000–99,999), and urban (≥100,000). Each locality was ranked using a social deprivation index (SDI) determined by its unemployment rate, median household income, the percentage of high school graduates in the adult population, and the percentage of blue‐collar workers in the active population 16. This SDI was previously validated in France as a tool for evaluating socioeconomic disparities in health at the municipality level 16. Localities divided into quartiles represented four classes: most deprived, deprived, privileged, and most privileged 16.

### Costs

The analysis was limited to direct costs, including those of drugs, drug administration, supportive care, and adverse events. Drug costs and their administration were based on national tariffs for DRGs and national fees for outpatient care. Cost data are expressed in 2016 euros (€).

### Statistical analyses

Data are descriptive. Continuous data are expressed as mean ± standard deviation (SD) or median (95% confidence interval [CI]), and categorical data as number (%). Survival rates by locality class and SDI were compared with hazard ratios (HRs) after adjustment for age, sex, and co‐morbidities. In the first step, univariate associations between incidence or survival and each variable‐of‐interest, were assessed individually using the *χ*² test (significance level, 0.05). In the second step, variables associated with the univariate model (*P *<* *0.20) were entered into a multivariate Cox model (stepwise selection with backward elimination; threshold, 0.05). Mortality rates were evaluated using Kaplan–Meier survival curves. Statistical Analysis System software, version 9.2 for Windows (SAS Institute Inc., Cary, NC) was used to compute all analyses.

### Ethics

The study was conducted in accordance with International Society for Pharmacoepidemiology (ISPE) Guidelines for good pharmacoepidemiology practices (GPP) and applicable regulatory requirements. Because this was a retrospective study using an anonymized database and had no influence on patient care, ethics committee approval was not required. The authorization number for administrative access to the DRG database was 2015‐111111‐56‐18 and the command numbers for the databases used were M14N056 and M14L056.

## Results

This analysis enabled identification of 1,890 new patients diagnosed with MPM during the 2‐year inclusion period (Table 1). The majority (76%) were men, whose mean age at diagnosis was 73.6 ± 10.0 years; 66% were >70 years old; 84% had at least 1 co‐morbidity. The initial diagnosis was most often made in general hospitals (51%), and more rarely in university hospitals (26%), or private clinics (24%). Compared to women, the men had significantly more frequent diabetes, renal insufficiency, respiratory insufficiency, or COPD. Patients usually lived in rural/semi‐rural areas and most deprived/deprived zones. All the patients were followed at least 2 years.

**Table 1:** Characteristics of the MPM patients

Characteristic	Total *n *=* *1,890	Men *n *=* *1,428	Women *n *=* *462	*P*
Age at diagnosis, years				
≤55	80 (4)^a^	48 (3)	32 (7)	<0.05
56–65	309 (16)	242 (17)	67 (15)	
66–75	601 (32)	455 (32)	146 (32)	
76–85	715 (38)	548 (38)	167 (36)	
>85	185 (10)	135 (9)	50 (11)	
At least 1 co‐morbidity among	**1584 (84)**	**1233 (86)**	**351 (76)**	**<0.0001**
Hypertension	812 (43)	626 (44)	186 (40)	NS
Diabetes mellitus	309 (16)	258 (18)	51 (11)	<0.001
Renal insufficiency	199 (11)	167 (12)	32 (7)	<0.01
COPD	199 (11)	175 (12)	24 (5)	<0.0001
Pulmonary insufficiency	416 (22)	330 (23)	86 (19)	<0.05
Other chronic lung diseases	1,305 (69)	1,021 (71)	284 (61)	<0.0001
Specific management				
Chemotherapy	1,235 (65)	931 (65)	304 (66)	NS
Curative surgery	14 (1)	10 (1)	4 (1)	NS
Population‐density index				
Rural	536 (28)	413 (29)	123 (27)	NS
Semi‐rural	535 (28)	415 (29)	120 (26)	
Semi‐urban	628 (33)	470 (33)	158 (34)	
Urban	185 (10)	126 (9)	59 (13)	
Undefined	6 (0)	4 (0)	2 (0)	
Social deprivation index				
Most deprived	513 (27)	381 (27)	132 (29)	NS
Deprived	475 (25)	366 (26)	109 (24)	
Privileged	398 (21)	308 (22)	90 (19)	
Most privileged	495 (26)	366 (26)	129 (28)	
Undefined	9 (0)	7 (0)	2 (0)	

NS, no significate.

a Values are expressed as *n* (%).

Treatment was purely symptomatic for 34% of the patients; 65% received at least one chemotherapy cycle (in 72% of cases pemetrexed alone or combined with bevacizumab). No disparity was observed concerning chemotherapy use according to the population‐density index and to SDI. Only 14 (1%) patients underwent curative surgery; 87 (5%) patients required intensive care at least once.

Analyze of the patient who died during the 2 years of follow‐up (Table 2), during the 3 months preceding deaths, 42% of the patients received at least one chemotherapy cycle (pemetrexed and/or bevacizumab for 47%), and 6% required intensive care at least once; during the last month of life, the respective rates were 20% (43% with pemetrexed and/or bevacizumab) and 5%.

**Table 2:** Descriptive analysis of 2‐year survival

Parameter	Total*n* (%)	2‐year survival		*P*
		Yes	No	
		*n* (%)	*n* (%)	
Patients, *n*	1,881	910 (48)	971 (52)	
Age, years				
≥70	633 (34)	316 (35)	317 (33)	NS
>70	1,248 (66)	594 (65)	654 (67)	
Sex				
Men	1,421 (76)	653 (72)	768 (79)	<0.001
Women	460 (24)	257 (28)	203 (21)	
Hypertension				
No	1,073 (57)	527 (58)	546 (56)	NS
Yes	808 (43)	383 (42)	425 (44)	
Diabetes				
No	1,573 (84)	783 (86)	790 (81)	<0.01
Yes	308 (16)	127 (14)	181 (19)	
Renal insufficiency				
No	1,682 (89)	840 (92)	842 (87)	<0.0001
Yes	199 (11)	70 (8)	129 (13)	
COPD				
No	1,683 (89)	830 (91)	853 (88)	<0.05
Yes	198 (11)	80 (9)	118 (12)	
Pulmonary insufficiency				
No	1,467 (78)	792 (87)	675 (70)	<0.0001
Yes	414 (22)	118 (13)	296 (30)	
Other chronic lung diseases				
No	583 (31)	329 (36)	254 (26)	<0.0001
Yes	1,298 (69)	581 (64)	717 (74)	
Population density of township				
Rural/semi‐rural	1,071 (57)	551 (61)	520 (54)	<0.01
Semi‐urban/urban	810 (43)	359 (39)	451 (46)	
Social deprivation of township				
Most deprived/deprived	988 (53)	469 (52)	519 (53)	NS
Privileged/most privileged	893 (47)	441 (48)	452 (47)	

NS, no significate.

Respective 1‐ and 2‐year survival rates were 64% and 48%. Median overall survival (OS) was 14.9 (95% CI: 13.7–15.7) months: significantly longer for women (18.2 [95% CI: 15.1–21.7] months) than men (14.1 [95% CI: 13.2–15.3] months; log‐rank test: *P *<* *0.001) and patients<70 years old (18.0 [95% CI: 15.7–20.0] months) than those older (13.3 [95% CI: 12.6–14.5] months; log rank test: *P *<* *0.0001).

Multivariate analyses retained male sex, advanced age (>70 years), chronic renal insufficiency, and chronic pulmonary insufficiency as predictive of poor outcome at 2 years (Table 3). Living in a rural/semi‐rural area appeared to be associated with a good outcome compared to those living in semi‐urban/urban areas, even after adjusting for age, sex, and co‐morbidities (HR: 0.83 [95% CI: 0.73–0.94] *P *<* *0.01). In contrast, SDI had no impact on survival.

**Table 3:** Univariate and multivariate comparisons of survival rates by commune class and Social Deprivation Index

Parameter	Univariate analysis			Multivariate analyses					
	HR	95% CI	*P*	No adjustment			Adjustment to age, sex, co‐morbidities		
				HR	95% CI	*P*	HR	95% CI	*P*
Age, years									
≥70	1.00	–	<0.0001	1.00	–	<0.0001	1.00	–	<0.0001
>70	1.41	1.23–1.61		1.40	1.23–1.61		1.40	1.22–1.61	
Sex									
Men	1.00	–	<0.001	1.00	–	<0.01	1.00	–	<0.01
Women	0.77	0.66–0.90		0.78	0.67–0.91		0.78	0.67–0.92	
Hypertension									
No	1.00	–	<0.15	1.00	–	<0.05	1.00	–	<0.01
Yes	0.90	0.80–1.03		0.85	0.75–0.97		0.84	0.84–0.96	
Diabetes									
No	1.00	–	<0.05			NS^b^	1.00	–	NS
Yes	1.21	1.03–1.42					1.12	0.95–1.33	
Renal insufficiency									
No	1.00	–	<0.01	1.00	–	<0.05	1.00	–	<0.05
Yes	1.30	1.08–1.56		1.27	1.06–1.54		1.27	1.05–1.53	
COPD									
No	1.00	–	NS	a			1.00	–	NS
Yes	1.09	0.90–1.32					0.95	0.78–1.16	
Pulmonary insufficiency									
No	1.00	–	<0.0001	1.00	–	<0.0001	1.00	–	<0.0001
Yes	1.75	1.53–2.01		1.71	1.49–1.96		1.70	1.47–1.97	
Other chronic lung diseases									
No	1.00	–	<0.10			NS	1.00	–	NS
Yes	1.15	1.00–1.33					0.99	0.85–1.16	
Population density of township									
Rural/semi–rural	0.82	0.73–0.93	<0.01	0.83	0.73–0.94	<0.01	0.83	0.73–0.94	<0.01
Semi‐Urban/urban	1.00	–		1.00	–		1.00	–	
Social deprivation of township									
Most deprived/deprived	1.11	0.98–1.26	<0.20			NS^b^			NS^b^
Privileged/most privileged	1.00	–							

NS, non‐significant; HR, hazard ratio; 95% CI, 95% confidence interval.

a Variable NS in the univariate analysis (*P* threshold = 0.20), therefore not included in the multivariate analysis.

b Variable NS (*P* threshold = 0.05) in the multivariate analysis, therefore removed from model.

A survival analysis restricted to patients whose entire chemotherapy regimen was administrated in the public sector (information on the type of chemotherapy is not available in the private sector) showed 1‐ and 2‐year survival rates of 72% and 52%, respectively and a median OS of 18.2 (95% CI: 17.0–19.5) months. Median OS for pemetrexed‐treated patients was significantly longer than for those not given it (18.5 [95% CI: 17.2–20.3] vs. 7.5 [95% CI: 5.3–15.3], respectively; *P *<* *0.0001); after adjustment for age, sex, and co‐morbidities, patients not given pemetrexed had a higher risk of death at 2 years than pemetrexed‐treated patients (HR: 2.39 [95% CI: 1.69–3.40]; *P *<* *0.0001).

The mean cost of managing this MPM‐patient cohort, from the perspective of health insurance, was €27,624 ± 17,263; costs of pemetrexed, and bevacizumab accounted for 31% of that expenditure.

## Discussion

This national MPM cohort analysis showed respective 1‐ and 2‐year survival rates of 64% and 48%, with median OS at 14.9 months. Men, older patients and those with co‐morbidities had poorer prognoses. These outcomes are better than those usually reported 17, 18. In a U.K. National Lung Cancer Audit 19 analyzing 8,740 cases seen in English and Welsh hospitals, median OS was 9.5 months, with respective 41.4% and 12.0% 1‐ and 3‐year survival rates. Median OS also varied by cancer network, increasing from 9.2 months in 2008 to 10.5 months in 2012.

For another English cohort of 910 patients, median OS was 10.0 months and an analysis dependent on the year of the treatment of new patients showed that those receiving pemetrexed‐based chemotherapy survived longer 20. In an Italian study, analyzing 241 MPM patients >70 years old, median OS was 11.4 months 21; age >75 years was associated with shorter OS. The role of age as a prognostic factor was also identified in a cohort of a1, 353 MPM patients diagnosed between 2005 and 2008 22. Their 1‐, 2‐, and 3‐year survival rates were 47%, 20% and 15%, respectively, and older age was independently associated with shorter survival (HR: 1.04 per year). Herein, median OS for patients >70 and >75 years, respectively, were 13.3 [12.6–14.5] and 11.4 [10.2–12.6] months.

Authors of one study reported that women with MPM survived longer than men whereas others have not 20, 21, 22. Analysis of the Surveillance, Epidemiology and End Results (SEER) database 23 showed that despite similar baseline characteristics for both sexes, 5‐year survival was 13.4% for women and 4.5% for men (*P *<* *0.0001). Even when adjusted for age, stage, race, and treatment, female MPM patients survived longer than men (HR: 0.78 [95%CI: 0.75–0.82]). In Ireland 24, median MPM‐patient survival was 6.5 months for men and 8.3 months for women. In our analysis, men had significantly more co‐morbidities, but after adjustment for age, sex, and co‐morbidities, being female remained a favorable prognosis factor for 2‐year survival. After adjusting analysis to age, sex and co‐morbidities, SDI did not influence survival. However, living in a rural/semi‐rural area was associated with significantly longer survival at 2 years, but we have no clear explanation for this observation.

Only 1% of the analyzed MPM population underwent curative surgery, a much lower rate than previously published 25, 26, but in good agreement with practices in France, where surgery is reserved for selected cases. These findings differ considerably from those of SEER‐database analyses, but we only considered potentially curative surgery and excluded palliative interventions. A1990–2004 SEER‐database analysis found a 22% rate of MPM‐directed surgery and significant predictors of undergoing such an intervention included race, age, and stage 26; median OS was 7 months. Multivariate analyses retained surgical treatments as independent predictors of longer survival 23. However, in a recent retrospective analysis of 1365 consecutive MPM patients, treated from 1982 to 2012 in six institutions, median OS for patients given palliative treatment or chemotherapy alone, pleurectomy/decortication or extra‐pleural pneumonectomy did not differ significantly and the authors concluded that the post‐surgical benefit was modest. A more recent study using a US National Cancer Database 27 evaluated survival after the treatment of MPM with cancer‐directed surgery. Stratified analysis revealed that surgery‐based multimodality therapy was associated with improved survival and may offer therapeutic benefit but only in carefully selected patients.

Use of chemotherapy varied substantially from one healthcare system to another 28: in our study, 67% received at least one chemotherapy cycle, a rate higher than that usually reported. Only 30% of the 8740 patients seen in English and Welsh hospitals received chemotherapy 19, and 36% in a population‐based study in Europe during a similar period 29. Pemetrexed use was associated with improved OS 21. In a cohort of 910 patients, 41% of whom received chemotherapy, median OS was 10.0 months and analyses dependent on the year of treatment of new patients showed that pemetrexed‐based chemotherapy recipients had longer survival 20.According to our analyses, not having been given pemetrexed was significantly associated with shorter survival. We did not find any disparity concerning chemotherapy use as a function of the area where the patient was living or the SDI, unlike studies on lung‐cancer management that found significant associations between outcomes and SDI 30. The short survival of PNM and lack of a definitive therapy may explain the absence of any potential differential effect due to SDI or other social characteristics.

Relatively little has been published worldwide specifically on the medical costs of treating MPMs 31, 32. Herein, the mean cost per patient was €27,624 ± 17,263. That value should be interpreted taking into account the characteristics of the French healthcare system which provides excellent management of medical care. A recent World Health Organization (WHO) study exploring MPM incidence, prevalence and costs for France calculated a mean cost of €15,900 per case. That estimate, below ours, can be explained, in part, by the recent availability of expensive chemotherapeutic agents that represented a third of the cost herein.

One of this study's limitations was the use of hospital databases. The assessment of the incident MPM cases (1,890 over a 2‐year period) was concordant with the national estimation (between 778 and 915 incident cases a year) 33 but database analysis did ‘not allow to assess the quality of life of these patients and also to take into account the indirect costs. MPM indirect costs represent a major part of the disease's economic impact. WHO estimated them to be €217 million for France in 2012, and that is probably an underestimation 34. In 2015, the fund to compensate asbestos victims (FIVA) 35 gave more than €120 million to MPM 514 patients, i.e., approximately €233,500 per person.

## Conclusion

With about 1000 incident cases per year in France, MPMs represent a significant burden for national healthcare system, with direct costs estimated at €27,624 per patient. Co‐morbidities, sex, age, and place of residence appear to be significant factors predicting the outcome, while SDI had no significant impact on survival.

## Conflict of Interest

None declared.
